# Hmx1 regulates *urfh1* expression in the craniofacial region in zebrafish

**DOI:** 10.1371/journal.pone.0245239

**Published:** 2021-01-19

**Authors:** Younes El Fersioui, Gaëtan Pinton, Nathalie Allaman-Pillet, Daniel F. Schorderet

**Affiliations:** 1 IRO – Institute for Research in Ophthalmology, Sion, Switzerland; 2 Faculty of Life Sciences, Swiss Federal Institute of Technology (EPFL), Lausanne, Switzerland; 3 Faculty of Biology and Medicine, University of Lausanne, Lausanne, Switzerland; Laboratoire de Biologie du Développement de Villefranche-sur-Mer, FRANCE

## Abstract

H6 family homeobox 1 (HMX1) regulates multiple aspects of craniofacial development as it is widely expressed in the eye, peripheral ganglia and branchial arches. Mutations in *HMX1* are linked to an ocular defect termed Oculo-auricular syndrome of Schorderet-Munier-Franceschetti (MIM #612109). We identified *UHRF1* as a target of HMX1 during development. UHRF1 and its partner proteins actively regulate chromatin modifications and cellular proliferation. Luciferase assays and *in situ* hybridization analyses showed that HMX1 exerts a transcriptional inhibitory effect on *UHRF1* and a modification of its expression pattern. Overexpression of *hmx1* in hsp70-hmx1 zebrafish increased *uhrf1* expression in the cranial region, while mutations in the *hmx1* dimerization domains reduced *uhrf1* expression. Moreover, the expression level of *uhrf1* and its partner *dnmt1* was increased in the eye field in response to *hmx1* overexpression. These results indicate that *hmx1* regulates *uhrf1* expression and, potentially through regulating the expression of factors involved in DNA methylation, contribute to the development of the craniofacial region of zebrafish.

## Introduction

Common to vertebrates, during early embryonic development, the neural crest cells, a transient migratory cell type, migrate from the dorsal neural tube to different regions of the body and contribute to the shaping of the developing embryo [1]. Cranial neural crest cells (NCC) from the developing forebrain and midbrain in respect to the eye development, give rise to the corneal endothelium, sclera, iris and ciliary body stroma [2], while hindbrain derived NCC play an important role in forming the branchial arches derivatives [3]. In zebrafish, migrating anterior cranial NCC surround cores of myogenic and vasculogenic mesoderm and form a series of pharyngeal arch primordia delimited by endodermal epithelial pouches [4]. The pharyngeal arches develop into several structures of the face and neck, including the maxillary and mandibular processes.

The development of the mammalian eye also requires the participation of two other embryonic tissue sources. The neural ectoderm gives rise to the neural retina, the pigmented epithelium and the optic stalk while cornea and lens originate from the surface ectoderm [5]. Morphogenetic transcription factors are involved in vertebrate eye development, including the H6 Family homeobox 1 (*HMX1*). *HMX1* belonging to a homeobox (*HMX*) family of transcription factors containing *HMX2* and *HMX3*, presents a phylogenetically conserved 60-amino acid homeobox domain [6].

In mouse, *Hmx1* is involved in the development of sensory neurons; it is highly expressed in the neural retina and peripheral ganglia as well as in the branchial arches [7]; moreover, schawnn cell precurors develop in consequence to a lack of neuronal specification mediated by Hmx1 in the neural crest ventral migratory pathway [8]. In zebrafish, *hmx1* is broadly expressed at 10-somite stage in the eye field, and from 24 hours post fertilization (hpf) is gradually restricted to the nasal side of the retina, before being limited to the nasal part of the inner nuclear layer. In the developing lens, *hmx1* is transiently expressed within a time window ranging from 24 to 72 hpf [9].

The molecular mechanisms regulating *HMX1* as well as the other eye field transcription factors are not yet fully understood and altered molecular signaling during early eye development may result in ocular malformation [10]. Schorderet et al. described a consanguineous family from Switzerland sharing a subset of ocular defects associated with a loss-of-function mutation in *HMX1* [11]. Microphthalmia, microcornea, cataract and ocular coloboma were described as part of a larger set of oculoauricular defects termed “Schorderet-Munier-Franceschetti” syndrome (OAS) (MIM: 612109) [11–14].

Two mutant mouse models called “*dmbo*” and “misplaced ears” exhibiting microphthalmia and ear and cranial malformations were reported. “*Dmbo*” mice showed malformations of the squamous temporal bone and hyperplasia of the gonial bone and failed to develop somatosensory neurons in the geniculate ganglion [7, 15]. In zebrafish, *hmx1* deficient embryos exhibited increased apoptosis in eye and brain in addition to a delayed withdrawal of retinal progenitors from cell cycle [9].

Despite the advances allowed by the use of several animal models, still not much is known about the role of *HMX1* in transcriptional regulation. Amendt et al. showed that HMX1 preferentially binds to a consensus sequence 5’-CAAGTG-3’ and acts as a transcriptional regulator [16]. Boulling et al. designed a predictive promoter model capable of screening *HMX1* potential target genes [17] and identified Ubiquitin-like, containing PHD and RING finger domains 1 (*UHRF1*).

DNA methylation is essential for epigenetic modulation of gene expression during mammalian development and has to be kept during cell division. The DNA daughter strand, after replication is methylated in accordance with the hemimethylated parent strand [18]. *UHRF1*, which contains a methyl DNA binding domain (SRA) binds preferentially to hemimethylated CpG sites and collaborates with DNA methyltransferase proteins DNMTs [19]. Changes in the methylation pattern, as in hypermethylation of CpG islands, result in gene silencing, whereas hypomethylation corresponds to gene transcription activation [20]. Uhrf1 has been implicated in the regulation of several developmental and homeostatic processes related to zebrafish development; the epigenetic modification mediated by *uhrf1* and its partner *dnmt1* is initiated at early embryonic stages where it is guiding the pre-gastrula development [21], while at later stages *uhrf1* is involved in both the regulation of intestinal development and the hepatic growth in zebrafish embryos [22, 23]. Zebrafish mutants of *uhrf1* and *dnmt1* showed altered lens development and maintenance [24], while limb mesenchymal cell-specific *Uhrf1* conditional knockout mice exhibit shortened long bones as a result of altered chondrocyte differentiation and proliferation [25].

In this study we identified *UHRF1* human promoter region and showed that HMX1 inhibits *UHRF1* expression in *in vitro* experiments. In situ hybridization in *hmx1* transgenic and mutant zebrafish showed that *uhrf1* expression is modulated in the hindbrain, eye region and branchial arches. Moreover, changes in *uhrf1* and *dnmt1* transcript levels in the eye field in heat shock transgenic zebrafish, indicate a possible involvement of hmx1 in regulating essential factors involved in DNA methylation.

## Materials and methods

### Plasmid contructions

A 1000-bp DNA amplicon around the transcription start site (TSS) of *UHRF1* carrying all the *HMX1*-binding sites identified was subcloned into the luciferase reporter pGL3-basic vector (Promega, Dubendorf, Switzerland) to produce the pGL3-Uhrf1 reporter construct for transfection in mammalian cells. The following primers were used: *UHRF1*-F 5’-TGCGAACGGACTTGGACTTA-3’ and *UHRF1*-R 5-CCTCCAAACCCTGGACCCT-3’. Human *HMX1* and mutant *HMX1* carrying deletions of the conserved domains SD1, SD2 and homeodomain were previously generated [26]. All DNA fragments were subcloned according to standard protocols.

### Cell culture and transfection

Human embryonic kidney (HEK) 293T cells were cultured in Dulbecco’s Modified Eagle’s Medium (DMEM) high glucose with stable glutamine (GE-Healthcare, Glattbrugg, Switzerland), supplemented with 10% FBS (Lonza, Basel, Switzerland), 100 U/ml penicillin and 100 μ/ml streptomycin (Invitrogen, Basel, Switzerland). Transfection occurred at approximately 50% confluence using the Calcium Phosphate method (ProFection Mammalian Transfection System, Promega, Dübendorf, Switzerland). The DNA ratio between pGL3 and pcDNA3 was held constant across transfections, which were repeated a minimum of 5 times.

### Luciferase reporter assay

48 hours post transfection the cells were harvest and firefly luciferase activity was measured using the DualGlo Luciferase Assay system (Promega, Dübendorf, Switzerland) on the Multiplate Reader Synergy HT (BioTek, Luzern, Switzerland) with KC4 software. The β-gal reporter under the control of a CMV promoter was used for normalization and transfections with stable β-gal values between the different conditions were considered.

### Statistical analysis

Average of the different experiments was expressed as mean ± sem.; either Student’s t-test, or Anova one way were used to express significance of difference between two groups. Significance was set at 0.05.

### Zebrafish maintenance and breeding

All animal procedures were carried out in accordance with the policies established by the Association for Research in Vision and Ophthalmology (ARVO) Statement for the Use of Animals and were approved by the Veterinary Service of the State of Valais (Switzerland). Zebrafish (Danio rerio) were maintained in a 14/10-h light/dark cycle; embryos were kept at 28.5°C in E3 medium [27]. All embryos at desired stages were kept in Danieau’s solution with 0.003% 1-phenyl-2-thiourea (Sigma, Buchs, Switzerland) to suppress pigmentation.

### Zebrafish-Tg (Hsp70-HMX1) line

The Tg(Hsp70-HMX1) transgenic line was previously generated and reported [26]. Embryos at desired stages were kept in Danieau’s solution with 0.003% 1-phenyl-2-thiourea (Sigma) to suppress pigmentation and heat-shocked for 30 min at 38°C. Four hrs post heat shock, embryos were euthanized and fixed for *in situ* hybridization underwent eye dissection procedure for RNA extraction and qRT-PCR analysis. Heat shock activation was confirmed by RT-PCR.

### Generation of zebrafish hmx1 mutant using zinc-finger nuclease (ZFN)

Zebrafish *hmx1* mutant was generated using zinc-finger nuclease (ZFN)-mediated mutagenesis. We identified zinc-finger nuclease (ZFN) target sites that were exclusively comprised within exon 2 of *Hmx1* (NM_001113526.1). Several zinc-finger arrays were designed against the target sites; cloning and ZFN mRNA synthesis followed standard protocols [28]; mRNA was injected into blastomeres of one-cell embryos. Hmx1 mutant zebrafish carrying the ZFN-deletion were confirmed by sequencing. Two groups of F0 zebrafish carrying single mutations named *Hmx1*^*mut10*^ and *Hmx1*^*mut150*^ were selected and separately raised to generate stable independent mutant lines. All experiments were carried with wildtype and mutant zebrafish collected at F6 and F7 generations.

### DIG-labeled RNA probes and whole-mount in situ hybridization

DIG-Labeled RNA probes were synthetized for *uhrf1* (NM_213077.1) with the following primers: *uhrf1*-F 5’-AGGATGGTCACACCATATTTG-3’ and *uhrf1*-R 5’-AGTTCACCATCACAATCATTCC-3’. In vitro transcription with the Roche RNA Labeling Kits (Roche diagnostics, Rotkreuz, Switzerland) was performed according to the manufacturer’s protocol. One-color whole-mount in situ hybridization was performed according to standard protocols [9]. Washing steps and antibody incubation were performed in the InSitu machine (BioLane HTI, Hölle&Hüttner, Tubingen, Germany).

### RNA extraction, cDNA synthesis and RT-PCR

For the analysis of *uhrf1* expression, wildtype and *hmx1* knockout embryos at 24 hpf and 5 dpf were euthanized. For whole body transcript analysis at 24 hpf and 5 dpf, 60 embryos obtained from different breeding were pooled together. For eye transcript analysis, both eyes were isolated from 20 embryos at 5 dpf and pooled together. All experiments were repeated three times.

First-strand cDNA synthesis was performed using the AffinityScript^™^ Multiple Temperature Reverse Transcriptase kit (Agilent, Basel, Switzerland) according the manufacturer’s protocol. cDNA was generated (GoScript Reverse Transcriptase System; Promega) and Real time PCR (FastStart SYBR Green Master Roche) was performed using *uhrf1*-F 5’-TCCAGGAGTCCAAGAGAGGAA-3’ and *uhrf1*-R 5’-TCTGCTGAACACAGTTCGGG-3’; *dnmt1*-F 5’-TTACTTTGGGCAAGAGGAGAGC-3’, *dnmt1*-R 5’-GACACCACACCGTTGTCTCT-3’; *hmx1*-F 5’-CGAAACCTCCAGGAG TCCAAG-3’, *hmx1*-R 5’-CGGGTCTTTTTCTTTCGGGC-3’.

Gene expression change was determined using the 2^–ΔΔCt^ method; relative values were normalized with β-actin.

## Results

### HMX1 regulates *UHRF1* expression in *in vitro* culture

**Fig 1:**
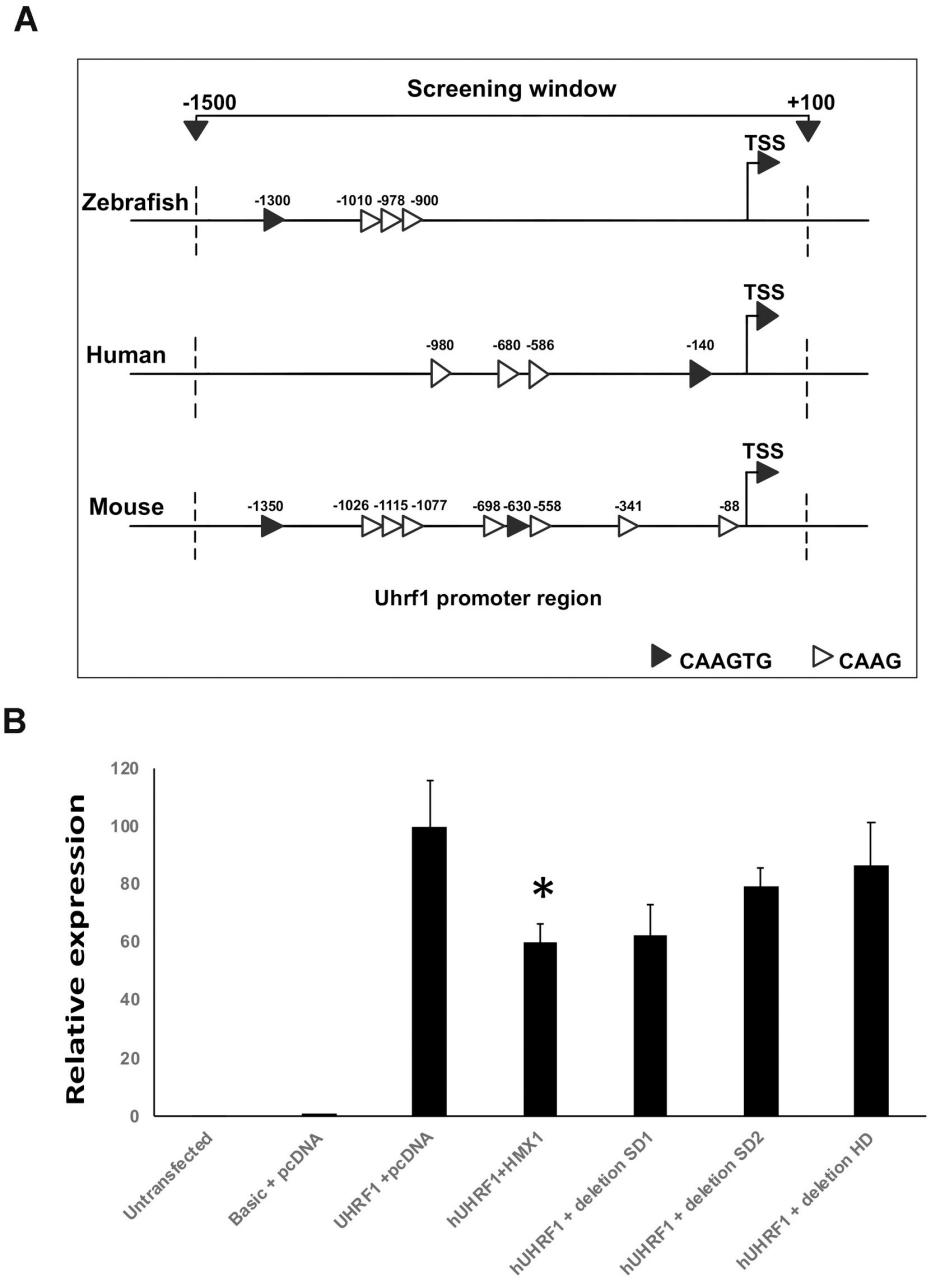
Schematic representation of the *HMX1* predicted promoter region in zebrafish, human and mouse genomes (A). Note the presence of complete Hmx1 binding sites (CAAGTG) and the minimal binding core (CAAG). Activity of HMX1 on human *UHRF1* promoter (B). The pGL3-Uhrf1 reporter plasmid was cotransfected with pcDNA3.1 empty, pcDNA3.1-Hmx1 expression vector, and pcDNA3.1-Hmx1 del SD1, del SD2, or del HD. HMX1 inhibits UHRF1 transcriptional activity by 40%. HMX1 del SD1, HMX1 del SD2 and HMX1 del HD had no statistical significant effect. Luciferase activity was normalized against β-galactosidase activity. Data are expressed as mean of three or more experiments. *; P<0.05 (ANOVA test).

We proceeded with identifying *UHRF1* promoter region using the Eukaryotic Promoter Database (https://epd.epfl.ch). In human, mouse and zebrafish genomes, inspection of a region ranging in size from −1500 to +100 nucleotides (nt) around the TSS showed the presence of both the canonical sequence 5’-CAAGTG-3’ and the minimal core motif 5’-CAAG-3’ with a lower affinity. The human region of interest contained one CAAGTG and three minimal CAAG motifs. In mouse, we observed two CAAGTG motifs and seven CAAG motifs, while in zebrafish one CAAGTG and three CAAG motifs were observed (Fig 1A).

To investigate the effect of HMX1 on *UHRF1* promoter *in vitro*, we co-transfected pGL3-UHRF1 and pcDNA3.1-HMX1 constructs. In presence of HMX1, we observed a decrease of 40% in *UHRF1* transcriptional activity (Fig 1B).

*HMX1* is composed of two exons with several conserved domains in exon 2: a homeobox (HD) and two domains SD1 and SD2, located 3´ to the HD [29]. The deletion of HD, SD1 or SD2 did not affect the inhibitory effect of HMX1 on *UHRF1* transcriptional activity as observed in luciferase assays (Fig 1B).

### *Uhrf1* is overexpressed in *hmx1* heatshock model

**Fig 2:**
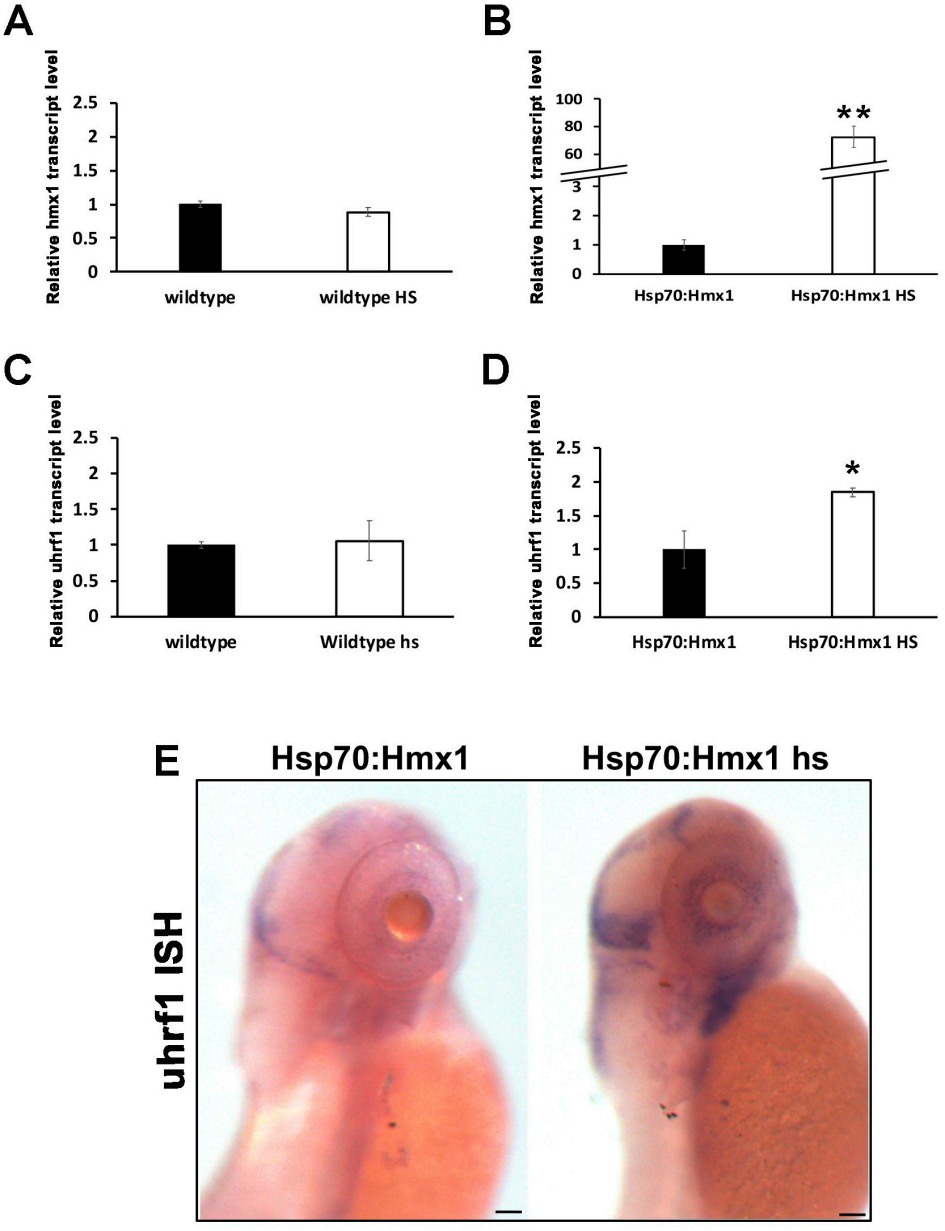
Expression of *Uhrf1* in hsp70:Hmx1 transgenic zebrafish at 5 dpf. (A-D) Quantitative RT-PCR from RNA isolated from zebrafish at 5 dpf. (A) Expression of *hmx1* in wildtype zebrafish without or with heat shock. (B) Expression of *hmx1* in transgenic zebrafish without or with heat shock. (C) Expression of *uhrf1* in wildtype zebrafish without or with heat shock. (D) Expression of *uhrf1* in transgenic zebrafish without or with heat shock. Heat shock treatment induced expression of Hmx1 in hsp70:Hmx1 but not in wildtype zebrafish. *Uhrf1* expression increased in hsp70:Hmx1 zebrafish embryos at 5dpf. (E) Lateral view of hsp70:Hmx1 zebrafish embryos at 5 dpf. In situ hybridization was performed with antisense RNA probe specific for *uhrf1*. *Uhrf1* expression increased in response to ubiquitous *hmx1* heat activation, but remained limited to the dorsal, eye and branchial arches regions. Bar, E 100 μm.

Wildtype zebrafish during development are highly enriched of *Uhrf1*, which is maternally provided up to the 5-prim stage embryos [23]; following the embryonic development, *uhrf1* is restricted to the cranial region and in particular to the lens and retina at 4 dpf. At 5 dpf uhrf1 is detected in both liver and gut. [24, 30] We designed a probe specific for *uhrf1* (S1 Fig) and showed that at 5 dpf, *uhrf1* is lightly expressed in the presumptive hindbrain, the eye field, the branchial arches (Fig 2E).

To investigate the regulation of *uhrf1*, we relied on induced activation of *hmx1* in a transgenic zebrafish model. We used a previously generated hsp70-hmx1 zebrafish transgenic line [26] to induce ubiquitous *hmx1* expression in response to a temperature stress condition to determine if the induction of the transgene would recapitulate the inhibitory effect we observed *in vitro*.

To discriminate between the potential effect of the temperature and *hmx1* overexpression on *uhrf1* expression, we subjected both wildtype and hsp70-hmx1 zebrafish to heat shock treatment at 5 dpf and compared them with their relative controls.

RT-PCR analysis on RNA isolated from heat shocked and control wildtype zebrafish did not detect any difference in the expression of *hmx1* and *uhrf1 (*Fig 2A and 2C). In hsp70-hmx1 zebrafish, overexpression of *hmx1* lead to increased *uhrf1* expression in comparison to control hsp70-hmx1 zebrafish as detected by RT-PCR analysis (Fig 2B and 2D). The *in situ* hybridization experiments revealed an increase of *uhrf1* expression which remained restricted to the presumptive hindbrain, the eye field and branchial arches (Fig 2E).

### *uhrf1* and *dnmt1* are overexpressed in the eye field of *hmx1* heatshock model

**Fig 3:**
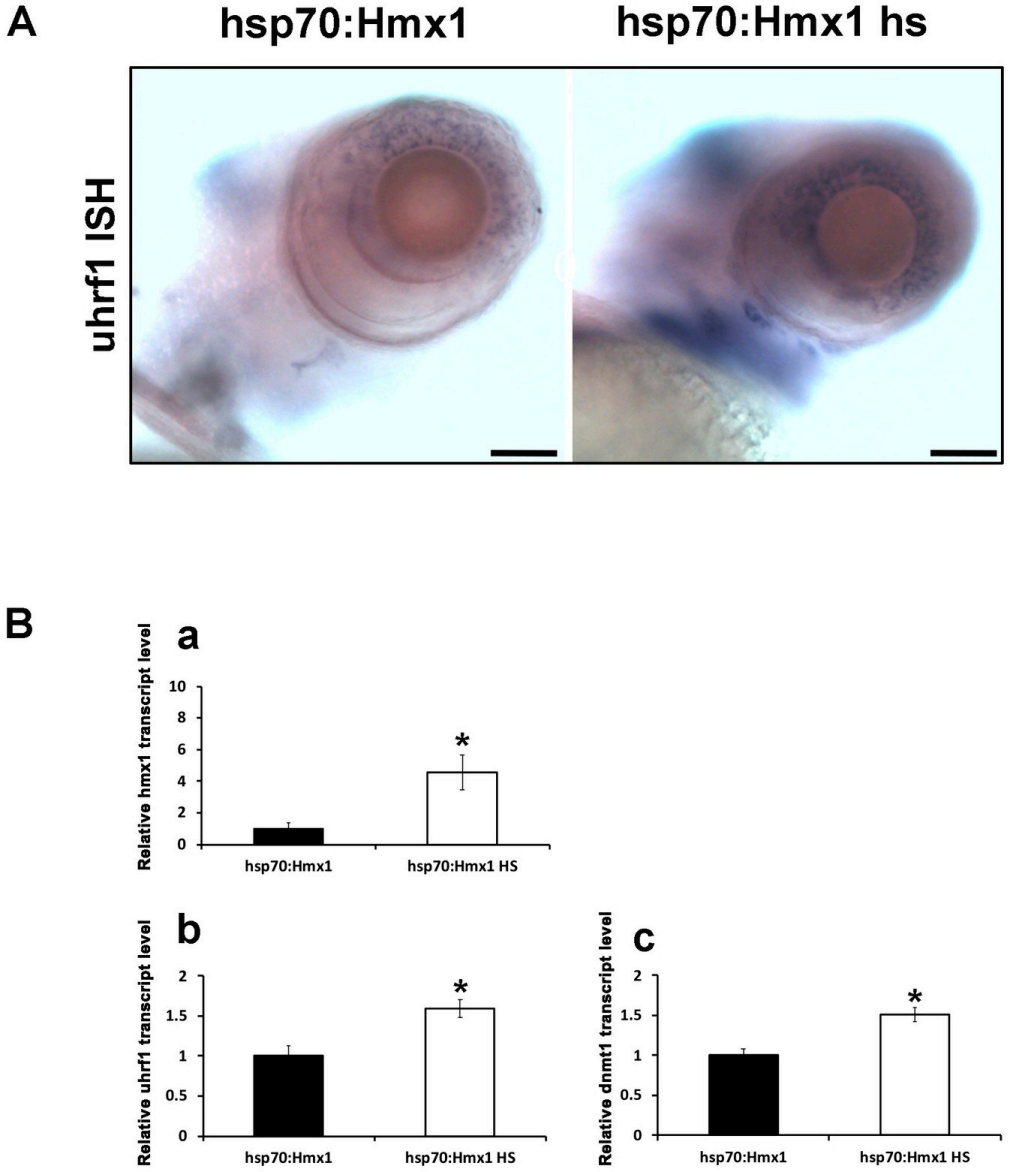
Lateral view of cranial region of hsp70:Hmx1 zebrafish embryos at 5 dpf. (A) *In situ* hybridization specific for *uhrf1*. *Hmx1* heatshock experiments induced *uhrf1* expression in the retina and branchial arches region. Quantification of RNA transcript isolated from zebrafish retinas at 5 dpf. (B) *Uhrf1* (b) and *dnmt1* (c) expression increased in response to the activation of *hmx1* in hsp70:Hmx1 embryos. Bar, A 75 μm.

It was previously reported that *uhrf1* and *dnmt1* are required for the development of the zebrafish lens [24]. To investigate whether Uhrf1 is involved in the development process of the retina, we analyzed the expression of *uhrf1* and *dnmt1* in the isolated eye region in control and heat shocked hsp70-hmx1 zebrafish at 5 dpf. In hsp70-hmx1 control zebrafish, *uhrf1* is expressed in the nasal part of the retina. In response to the overexpression of *hmx1*, *uhrf1* expression pattern changed, resulting in a spread localization towards the inner region of the retina (Fig 3A). To quantify the changes in the expression pattern, we performed quantitative RT-PCR analysis on RNA isolated from the eye field at 5 dpf and observed a 59% and 51% increase in *uhrf1* and *dnmt1* expression respectively (Fig 3B).

### Generation of *hmx1* zebrafish mutant

**Fig 4:**
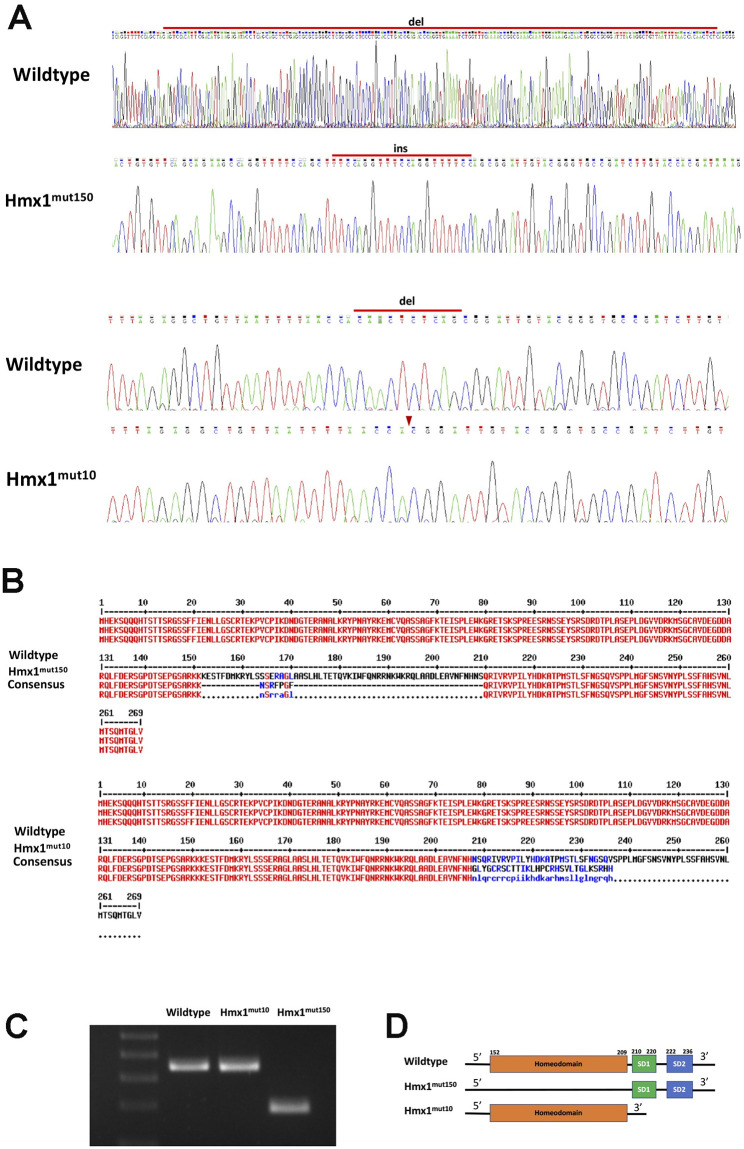
Hmx1 mutant zebrafish generation via zinc-finger nuclease method. (A) Wildtype and hmx1 mutant sequence analysis. Hmx1^mut150^ mutant embryos carried a 170 bp deletion and a 21 bp insertion, while Hmx1^mut10^ embryos reported a 10 bp deletion. (B,D) ExPasy translation tool depicted the disruption of the encoding sequence of the homeodomain and the downstream region composed of SD1 domains in exon2. C. Agarose gel of PCR amplified DNA products. The DNA isolated from WT, *hmx1*^*mut10*^ and *hmx1*^*mut150*^ embryos was amplified by PCR.

*HMX1* is composed of two exons, with three conserved domains in exon 2. The homeodomain (HD) and SD1 were implicated in the dimerization of HMX1. Mutations that removed the dimerization domains of Hmx1 inhibited Hmx1 activity on potential targets [26]. We used (ZFN)-mediated mutagenesis to generate mutations in *hmx1*. Several mutations were screened and two mutants were selected. *Hmx1*^mut150^ zebrafish carried a deletion of 150 nucleotides, replaced with an insertion of 21 base pairs while *hmx1*^mut10^ presented a deletion of 10 base pairs. The deletions were confirmed by electrophoresis and sequencing analysis. Using ExPasy translation tool, we verified that the mutations disrupted the encoding sequence of the homeodomain and the downstream region including SD1 and SD2 domains in exon2 (Fig 4). The *Hmx1*^mut150^ had an indel mutation that replaced part of the homeodomain with a 7-amino acid unrelated sequence, but left intact the SD1 and SD2 domains while the *hmx1*^mut10^ had a deletion and a frameshift mutation generating a termination codon at position N380 (N380Qfs*30).

### *Hmx1*^*mut10*^ and *Hmx1*^*mut150*^ zebrafish have smaller eyes and a partial aberrant lens phenotype

**Fig 5:**
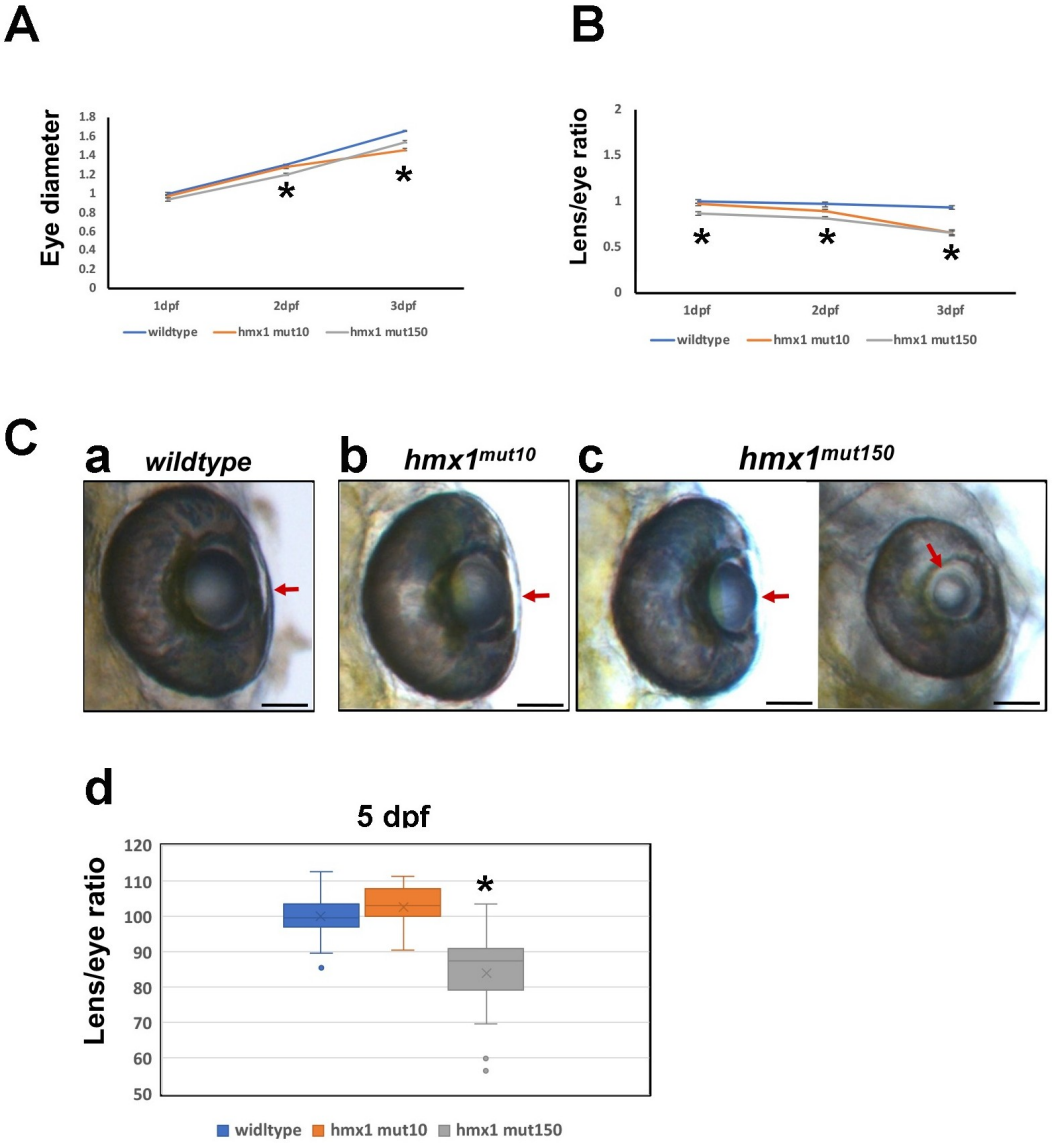
Morphometric analysis of the developing zebrafish eye at early stages. (A) Tracking of the growth of wildtype, *hmx1*^mut10^ and *hmx1*^mut150^ eye from 1 dpf to 3 dpf. *Hmx1*^mut10^ and *hmx1*^mut150^ eyes are reduced in size compared to wildtype embryos at 2 and 3 dpf stages. (B) Lens growth expressed as ratio lens/whole eye. *hmx1*^*mut10*^ and *hmx1*^*mut150*^ lenses are smaller at 3 dpf. (C) Wildtype, *hmx1*^*mut10*^ and *hmx1*^*mut150*^ eyes at 5 dpf. Wildtype and *hmx1*^*mut10*^ present a fully developed lens (5C, a-b), while *hmx1*^*mut150*^ embryos exhibit two opposing phenotypes (5C, c); on the left side a recovered lens is presented, while on the right a smaller lens presenting a clear phenotype called microphakia. (5C, d) graphic representation of lens/eye ratio; *hmx1*^*mut150*^ presents a wide range of distribution of the lens size. Red arrows indicate the lens at 5 dpf. Data are expressed as mean of three to 5 experiments. *; P<0.05 (ANOVA test). Bar, C 100 μm.

Following the generation of zebrafish *Hmx1*^*mut10*^ and *Hmx1*^*mut150*^, we performed a morphometric analysis of the developing eye. Three parameters were considered: diameter of the eye, lens size and its ratio. By tracking wildtype eye growth, starting at 1 dpf and reaching 3 dpf stage, we observed a continuous growth in size (Fig 5A). *Hmx1*^*mut10*^ eye size was identical to wildtype eye size at both 1 dpf and 2 dpf stages, while at 3 dpf *Hmx1*^*mut10*^ eye was smaller in a significant manner. *Hmx1*^*mut150*^ eye was significantly smaller at all considered time points, while still reflecting the same growth pattern observed for the wildtype eye development (Fig 5A). We then focused on the development of the lens in relation to the growth of the eye. We observed that the wildtype ratio lens/eye remained unchanged over time up to 3 dpf, indicating that the lens growth was directly proportional to the growth of the whole eye (Fig 5B). *Hmx1*^*mut10*^ lens/eye ratio at 1 dpf was similar to wildtype ratio, while at 2 dpf decreased and at 3 dpf indicated that *Hmx1*^*mut10*^ lens was significantly. *Hmx1*^*mut150*^ lens was smaller at all considered time points (Fig 5B). Both *Hmx1*^*mut150*^ and *Hmx1*^*mut10*^ lenses/eyes presented the same ratio.

At 5 dpf both eye and lens were fully developed in both wildtype and *Hmx1*^*mut10*^ embryos (Fig 5Ca and 5Cb), while in *Hmx1*^*mut150*^ zebrafish, we observed a recovered phenotype and a severe phenotype with a smaller lens (Fig 5Cc). The quantification of the ratio lens/eye confirmed the recovery of the lens growth for *Hmx1*^*mut10*^ embryos and that *Hmx1*^*mut150*^ zebrafish generally presents a smaller lens (Fig 5Cd).

### Reduced expression of *uhrf1* in *hmx1* zebrafish mutant

**Fig 6:**
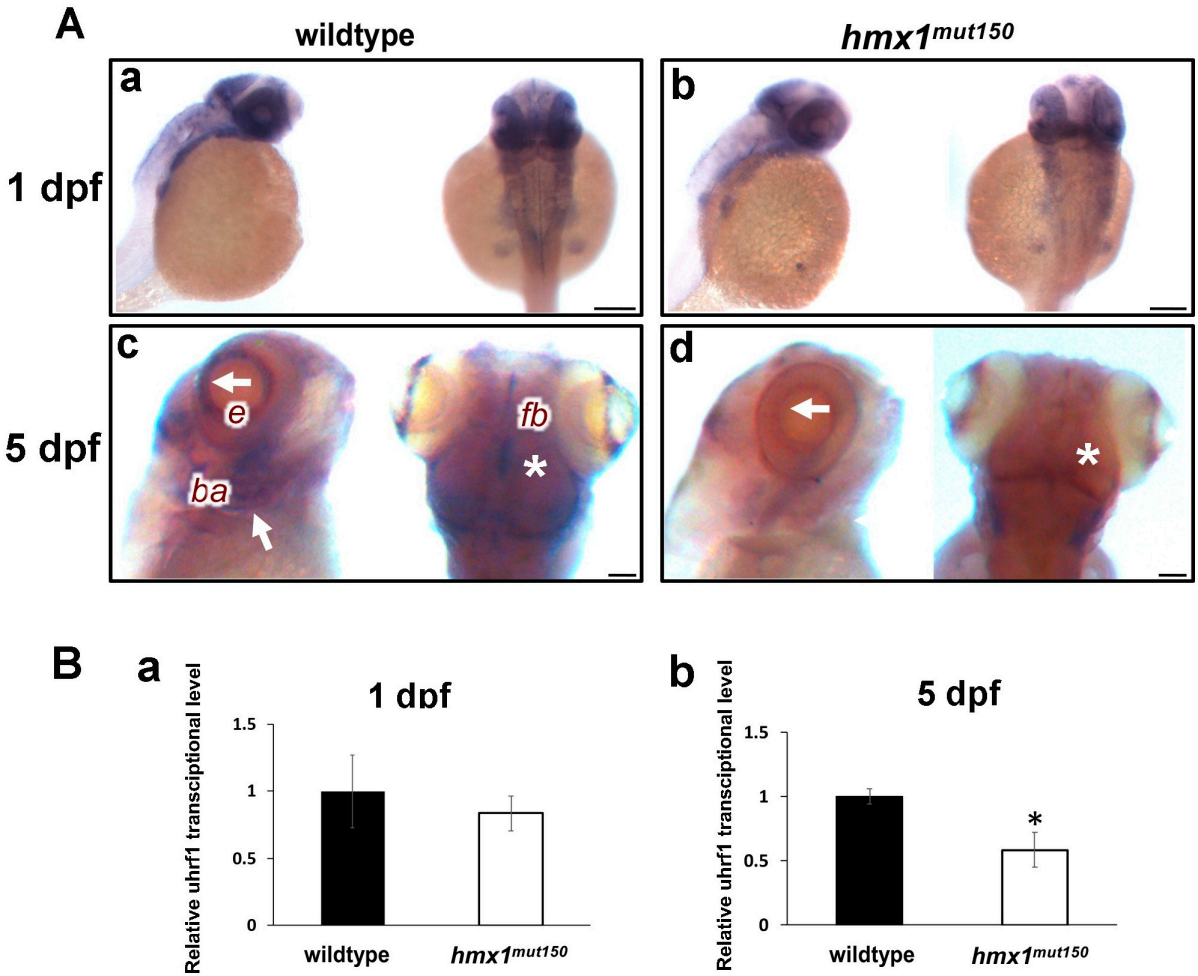
In situ hybridization specific for *uhrf1* in wildtype and *hmx1* mutant zebrafish at 1 dpf. (A) *uhrf1* is expressed in the dorsal region and in the eye field region at 1 dpf in both wildtype and hmx1 mutant embryos (a,b). c,d: *uhrf1* expression is reduced in the eye field, in the forebrain and branchial region (c,d). White arrow, retina and branchial arches; asterisk, forebrain. e, eye; fb, forebrain; ba, branchial arches. RT-PCR quantification of *uhrf1* transcript isolated from the cranial region. (B) *Uhrf1* expression is reduced at 5 dpf (b) but not at 24 hpf (a). Bar, A 100 μm.

We questioned whether the inhibition of *hmx1* in zebrafish would affect *uhrf1* expression. Both *hmx1*^mut150^ and *hmx1*^mut10^ embryos developed a smaller eye therefore the expression pattern of *uhrf1* was examined by in situ hybridization in wildtype and *hmx1*^mut150^ zebrafish during development. At 1 dpf, *uhrf1* mRNA is progressively acquiring a distinct and regionalized expression pattern and is highly expressed in the eye anlage, in the midbrain, the presumptive optic tectum and the caudal region of the hindbrain (Fig 6Aa). *In situ* hybridization and qRT-PCR analysis of *hmx1* mutants at 24 hpf did not show a significant alteration of *uhrf1* expression in comparison to wildtype embryos (Fig 6Ab and 6Ba).

In 5 dpf wildtype zebrafish, *uhrf1* transcript is detected mainly in the cranial region with an expression pattern limited to the retina, the midbrain and the branchial arches. *In situ* hybridization of zebrafish embryos carrying mutations in *hmx1* exhibited a distinctive phenotype characterized by a reduced expression and undetectable *uhrf1* signal in the developing branchial arches (Fig 6Ac and 6Ad); similarly *in situ* hybridization of *hmx1*^mut10^ embryos indicated a reduced expression for *uhrf1* in the cranial region (S1B Fig). RT-PCR analysis of the transcript isolated from the cranial region confirmed that *uhrf1* expression was significantly reduced in *hmx1* knockout zebrafish (Fig 6Ab).

## Discussion

The OAS of Schorderet-Munier-Franceschetti (MIM:612109) is caused by defects associated with mutations in the *HMX1* transcription factor [11]. *Hmx1* has been since investigated for its involvement in regulating the somatosensory organ genesis and development [7] as well as for its role in the neuronal fate of migrating neural cells [8, 31]. While mammals have a single *HMX1* gene, the teleost zebrafish has a duplication of *hmx1* located in a 1.2 kb distance: *hmx1* and *hmx4* previously known as *Soho1* [32].

In zebrafish, *in situ* hybridization revealed a transient *hmx1* expression in the developing lens between 24 and 72 hpf. Using a morpholino-based inhibition of *hmx1* translation, Boisset et al. observed microphthalmia and delayed retinogenesis at 3 dpf. At 5 dpf, retinal and lens formation recovered but microphthalmia persisted [9]. Morpholino inhibition experiments also showed that reduced *hmx4* expression causes severe neural defects ranging from failure of the neural tube closure to the narrowing of the eye field [22]. The discovery of *Hmx1* binding sequence 5’-CAAGTG-3’ and the developed predictive promoter model are important tools to identify potential downstream targets and exploring the morphogenetic aspects in which *Hmx1* could be involved.

*Uhrf1* expression is crucial for proper embryonic development as during oocyte growth stage *Uhrf1* contributes to CG methylation [33] while between 24 and 48 h after fertilization, *uhrf1* is highly expressed in proliferative tissues including the tectum, retina and branchial arches. At 4 and 5 dpf, *uhrf1* persists in liver bud and gut [30]. *Uhrf1* deficiency is reported to alter the epigenetic pattern of several target genes and consequently induce transcriptomic changes resulting in developmental abnormalities. Deletion of maternal *Uhrf1* causes developmental defects in preimplantation embryos [34], while at pre-gastrulation stage, altered *uhrf1* expression results in embryonic lethality [23]. Knockout of zebrafish *uhrf1* and *dnmt1* presented defects in lens formation and microphthalmia [24], while conditional *Uhrf1* deficiency in mouse model increased the expression of several genes including *Hspb1*, a gene involved in chondrocyte differentiation [35] and suppressed osteoblast activity [36].

Recently, the phenotype of OAS has been extended by the description of additional patients [12, 13]. The increasing number of patients reporting aberrant orofacial development is pointing to a possible major role for *HMX1* as part of a complex gene network regulating maxilla and mandibles formation. As a consequence of a perturbed *HMX1* activity short mandibular rami, asymmetry of the jaws and altered premaxilla were observed.

Zebrafish and mouse models mutated for *Hmx1* and *Uhrf1* presented similar altered phenotypes, suggesting that a functional interaction might be established between the two genes; we therefore investigated the relationship between *HMX1* and *UHRF1* (Fig 1A).

We showed *in vitro* that HMX1 exerts a transcriptional inhibitory effect on *UHRF1* and that the complete form is necessary for HMX1 activity since mutant constructs carrying HD, SD1 or SD2 deletions did not show significant inhibitory effects on *UHRF1* (Fig 1B). This is in accordance with previous work showing that HMX1 activity is linked to its dimerization and presence of these structures [26].

We consequently investigated the causative relationship between *hmx1* and *uhrf1* in zebrafish and the effect of overexpression and inactivation of *hmx1* during embryonic morphogenesis.

Whole mount in situ hybridization and quantitative RT-PCR analysis in the eye field showed that *hmx1* overexpression and inactivation resulted in a modification of *uhrf1* expression pattern (Figs 2 and 3). At 1 dpf, overexpression and inactivation of *hmx1* did not affect *uhrf1* transcript in the eye field and generally at the level of the craniofacial region as *uhrf1* was still expressed in the developing mid and hindbrain features. A possible explanation could be that *uhrf1* mRNA is maternally provided and in sufficient amounts to allow the development during the first 24 hpf [21, 23], thus masking the possible *uhrf1* gene regulation at the 5 prim 24 h stage. Additionally, several factors are actively contributing to the methylation process during zebrafish early development [37].

At 5 dpf, *uhrf1* expression in *hmx1* knock-out was reduced or absent in the retina and branchial arches (Fig 6c and 6d). Moreover, in the ventral region *uhrf1* expression persisted in the gut, indicating that *uhrf1* is affected only in the cranial region where *hmx1* is normally expressed in physiological conditions (S1C Fig).

By using the zebrafish model, we noted that the mutated *hmx1* affected *uhrf1* expression in the derivatives of the cranial neural crest cells in the craniofacial region. Finally, we observed that *hmx1* negatively regulated *uhrf1* expression *in vitro*, while in zebrafish carrying mutations for *hmx1*, *uhrf1* expression was also reduced. Differences in experimental outcomes from *in vitro* and *in vivo* settings are often reported due to the nature of the systems utilized; while in *in vitro* cultures a controlled environment is a prerequisite, in *in vivo* experiments multiple independent factors are contributing to the regulation of the mechanism under investigation and this could possibly explain the different outcome we observed. Alternatively, the difference detected between the two systems could be explained by *hmx1* potential capability to exert both activating and repressive functions towards downstream targets as previous studies reported for other signaling factors [38, 39].

Moreover, a morphological analyis of the development of the eye of the newly generated *hmx1* mutant embryos, indicated that during early development, *Hmx1*^*mut10*^ and *Hmx1*^*mut150*^ developed a smaller eye (Fig 5). At 3 dpf, a stage crucial for lens development [9], both mutant zebrafish presented a smaller lens compared to wildtype embyros. At 5 dpf, while *Hmx1*^*mut10*^ presented a fully developed lens, in a subgroup of *hmx1*^*mut150*^ embryos a severe lens phenotype called microphakia persisted. The morphological defects related to lens development, are consistent with the altered *uhrf1* phenotype previously presented [24]. The recovery of the lens development we observed in *Hmx1*^*mut10*^ and partial recovery in *Hmx1*^*mut150*^ could be explained by the potential and complementary activity of *hmx4* at this critical stage for lens development.

Our work combined with studies from other groups shows that *Hmx1* could be part of a cluster of modulators regulating the expression and patterning of *Uhrf1*.

Further studies are needed to elucidate the contribution of both *Hmx1* and hmx4 during cranial development; here to discern between *hmx1* and *hmx4* functions in zebrafish, a comparative transcriptome analysis is an essential approach to detect differences in the regulatory mechanisms attributed to *hmx1* and *hmx4* genes. The investigation of the potential methylation process mediated by *uhrf1* of factors involved in eye formation could be resolved by the analysis of the changed methylation pattern of genes involved in retinal and lens development.

## Supporting information

S1 Fig (A) *In situ* hybdrization performed with antisense (A-C) and sense (B-D) probes. Uhrf1 expression was detected with the sense probe in the cranial region, eye and gut of wildtype zebrafish at 5 dpf. (B) *uhrf1* expression at 5 dpf in wildtype and *hmx1*^mut10^ zebrafish in the cranial region. (C) *uhrf1* expression at 5 dpf in wildtype and *hmx1*^mut150^ zebrafish in cranial and ventral regions. Bar, A 100 μm. White arrow head; gut.
(TIF)

S1 Raw images (TIF)
