# Supplementary figures and images for: Hmx1 regulates urfh1 expression in the craniofacial region in zebrafish
Source: PLoS One. 2021 Jan 19;16(1):e0245239. doi: 10.1371/journal.pone.0245239 (PMC7815118; doi:10.1371/journal.pone.0245239)

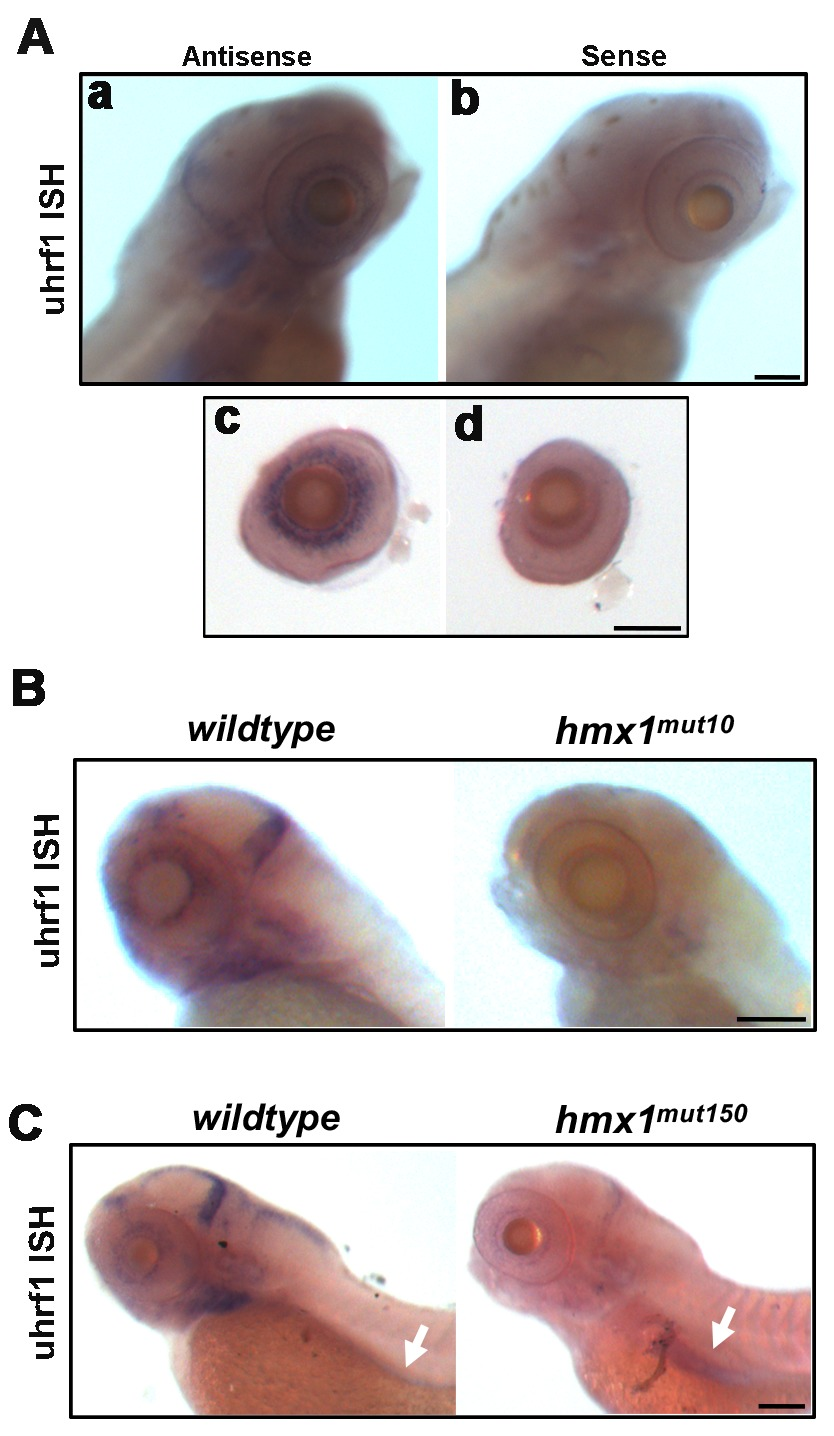

Supplement: S1 Fig — (A) In situ hybdrization performed with antisense (A-C) and sense (B-D) probes. Uhrf1 expression was detected with the sense probe in the cranial region, eye and gut of wildtype zebrafish at 5 dpf. (B) uhrf1 expression at 5 dpf in wildtype and hmx1mut10 zebrafish in the cranial region. (C) uhrf1 expression at 5 dpf in wildtype and hmx1mut150 zebrafish in cranial and ventral regions. Bar, A 100 μm. White arrow head; gut. (TIF) [file pone.0245239.s001.tif]

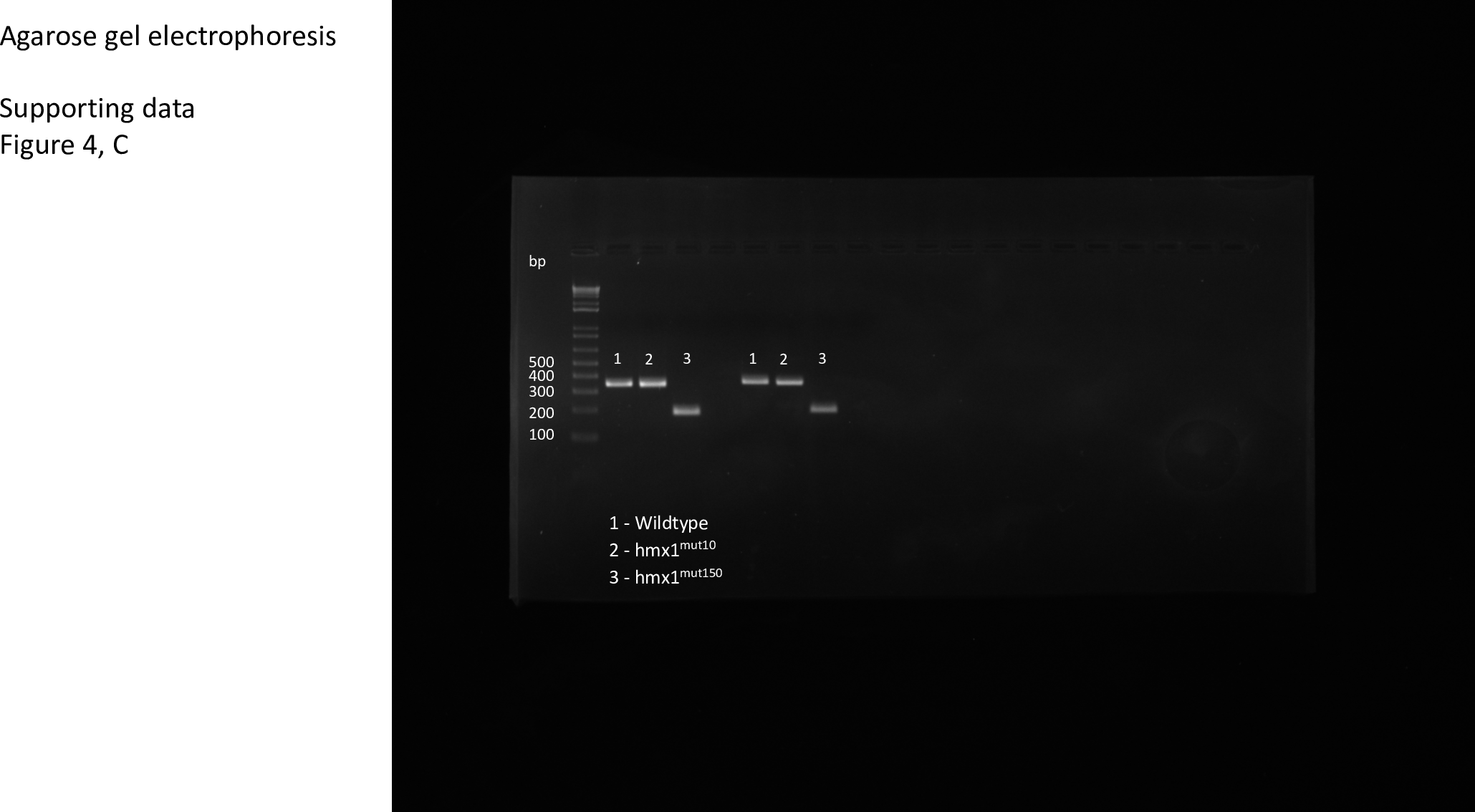

Supplement: S1 Raw images — (TIF) [file pone.0245239.s002.tif]
